# A False Positive I-131 Metastatic Survey Caused by Radioactive Iodine Uptake by a Benign Thymic Cyst

**DOI:** 10.1155/2017/6469015

**Published:** 2017-12-20

**Authors:** Avneet K. Singh, Adina A. Bodolan, Matthew P. Gilbert

**Affiliations:** ^1^Department of Medicine, The Robert Larner, M.D. College of Medicine at The University of Vermont, Burlington, VT, USA; ^2^Department of Pathology and Laboratory Medicine, The Robert Larner, M.D. College of Medicine at The University of Vermont, Burlington, VT, USA; ^3^Division of Endocrinology and Diabetes, The Robert Larner, M.D. College of Medicine at The University of Vermont, Burlington, VT, USA

## Abstract

Thyroid carcinoma is the most common endocrine malignancy in the United States with increasing incidence and diagnosis but stable mortality. Differentiated thyroid cancer rarely presents with distant metastases and is associated with a low risk of morbidity and mortality. Despite this, current protocols recommend remnant ablation with radioactive iodine and evaluation for local and distant metastasis in some patients with higher risk disease. There are several case reports of false positive results of metastatic surveys that are either normal physiologic variants or other pathological findings. Most false positive findings are associated with tissue that has physiologic increased uptake of I-131, such as breast tissue or lung tissue; pathological findings such as thymic cysts are also known to have increased uptake. Our case describes a rare finding of a thymic cyst found on a false positive I-131 metastatic survey. The patient was taken for surgical excision and the final pathology was a benign thymic cyst. Given that pulmonary metastases of differentiated thyroid cancer are rare, thymic cysts, though also rare, must be part of the differential diagnosis for false positive findings on an I-131 survey.

## 1. Case Description

**Figure 1:**
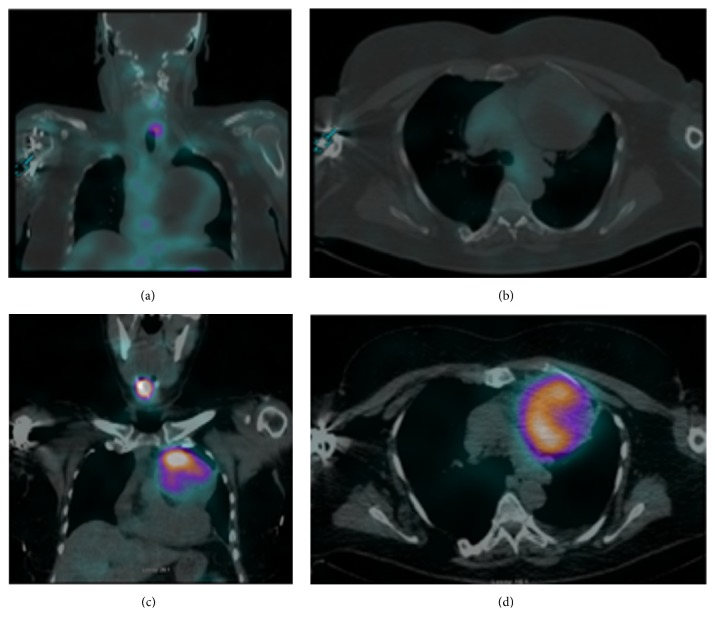
An initial diagnostic SPECT/CT scan was performed 24 hours after administration of 5.3 mCi of Iodine-123. There is uptake in the left, posterior thyroid bed and anteriorly at the level of the hyoid bone. Physiologic uptake was noted in the salivary glands, nasopharyngeal mucosa, and gastrointestinal tract. A large (8 cm × 9 cm × 8 cm) heterogeneous left anterior mediastinal mass with mixed solid and cystic architecture was noted. This mass did not demonstrate uptake of the Iodine-123 tracer ((a), (b)). Posttreatment SPECT/CT one week later showed uptake of radioactive iodine by the mediastinal mass ((c), (d)).

A 61-year-old female was found to have a palpable thyroid nodule on routine physical examination by her primary care physician. The patient was then referred for a thyroid ultrasound which showed a 2.1 cm, left-sided thyroid nodule. The patient underwent an ultrasound guided fine needle aspiration (FNA) biopsy which revealed a papillary thyroid carcinoma. The patient was referred to ENT and subsequently had a total thyroidectomy. She was referred to our endocrine clinic for postsurgical management of her T1b N1a MX stage 3 papillary thyroid carcinoma 4 weeks after total thyroidectomy. The tumor size was 1.7 cm in maximum diameter and was unifocal. At the time of surgery, the surgical margins were positive and focal lymphovascular invasion was observed with 1 out of 6 dissected lymph nodes positive for metastatic disease. The patient was prepared for radioactive iodine remnant ablation with a low iodine diet and thyrotropin alfa injections. An initial diagnostic SPECT/CT (Philips Precedence 16P) scan was performed 24 hours after administration of 5.3 mCi of Iodine-123 (I-123). The scan showed uptake in the left posterior thyroid bed and anteriorly at the level of the hyoid bone, both likely representing residual thyroid tissue. Physiologic uptake was also noted in the salivary glands, nasopharyngeal mucosa, and gastrointestinal tract. Additionally, a large (8 cm × 9 cm × 8 cm) heterogeneous left anterior mediastinal mass with mixed solid and cystic architecture was noted. This mass did not demonstrate uptake of the I-123 tracer (Figures 1(a) and 1(b)). A CT-guided biopsy of the mass returned only hemorrhagic material. Serum thyroglobulin tumor marker was found to be low at 0.3 ng/dL and TSH was >125 uIU/mL, which suggested the mass was not metastatic thyroid cancer. Based on these laboratory findings, it was decided to continue with ablation therapy and 157 mCi of Sodium Iodide-131 (I-131) was administered to the patient. Posttreatment SPECT/CT one week later showed uptake of radioactive iodine by the mediastinal mass (Figures 1(c) and 1(d)). After a repeat CT-guided biopsy was nondiagnostic, the mass was removed via sternotomy.

**Figure 2:**
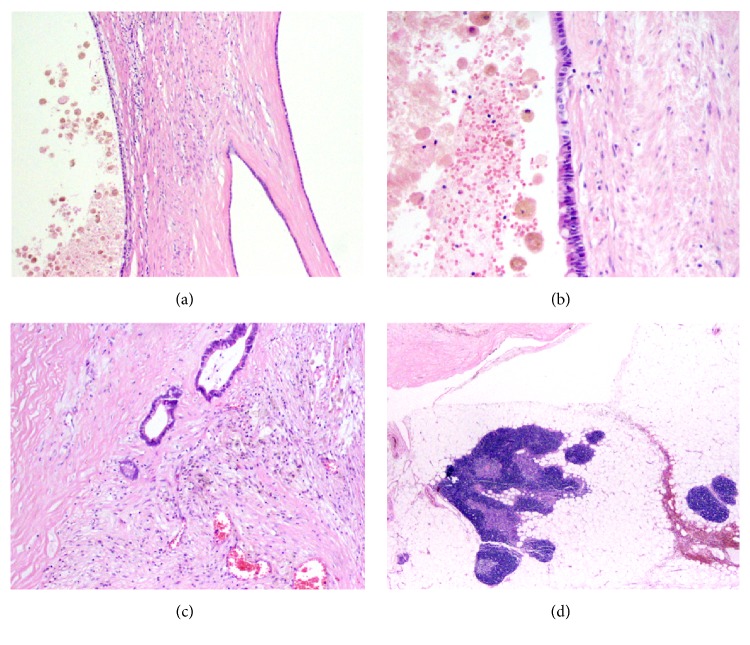
Surgical pathology sections of 9.0 cm multilocular thymic cyst. (a) Cyst walls lined by simple low-cuboidal epithelium. (b) Cyst wall lined by simple columnar mucinous epithelium with goblet cells. (c) Fibrous stroma with glandular elements and numerous hemosiderin-laden macrophages. (d) Remnant of normal involuting thymic tissue adjacent to cystic spaces.

Surgical pathology sections demonstrated a multiloculated mass composed of cystic spaces lined by a variety of epithelial types, including areas of low-cuboidal (Figure 2(a)), mucinous columnar with goblet cells (Figure 2(b)), ciliated columnar, and keratinizing stratified squamous epithelium. There was no significant cytological atypia or increased mitotic activity. The intervening stroma was densely fibrotic with patchy chronic inflammation, glandular elements, and numerous hemosiderin-laden macrophages—providing evidence of previous hemorrhage (Figure 2(c)). Grossly, the cystic contents ranged from dark brown, likely due to previous hemorrhage, to clear fluid. Adjacent to the cystic spaces, there was a peripheral remnant of normal involuting thymic tissue showing Hassall corpuscles and associated lymphoid tissue (Figure 2(d)). These findings are consistent with a benign multilocular thymic cyst.

## 2. Discussion

Thyroid carcinoma is a disease of significant interest in American public health given the increasing trend of diagnosis and incidence with relatively stable mortality [1]. Differentiated thyroid cancer (DTC) is associated with a low risk of morbidity and incidence of distant metastasis is relatively uncommon [2]. Recent statistics indicate patients diagnosed with DTC present with distant metastases in approximately 4.2% of cases [3]. As part of the thyroid carcinoma treatment and surveillance algorithm, postthyroidectomy surveillance and assessment for metastases are still commonly accomplished with I-131 metastatic surveys [4]; however, I-123 scans have increased resolution with improved quality of imaging, often characterizing masses missed on I-131 imaging [2].

Uptake of radioiodine by nonthyroid structures can result from normal expression of the sodium-iodide symporter (NIS), presence of metabolized or physiologically retained radioiodine in body fluids, uptake of radioiodine by inflamed tissues, or contamination. Major pathological mechanisms of increased I-131 uptake not related to metastases from thyroid carcinoma include those of pathological transudates, inflammation (acute or chronic), and neoplasms of nonthyroid origin [5]; nonpathological variants include tissues with increased secretions in addition to typical expression of NIS and iodine accumulation by lactating breast tissue [2]. Given the complex mechanisms of I-131 uptake, distinguishing between pathological and normal anatomic variants on metastatic survey can prove problematic. Thus, false positive imaging results from I-131 metastatic surveys can and do occur at a rate of 3.85% in patients with DTC [6].

In our case, initial findings on the I-131 posttherapy metastatic survey indicated the presence of possible metastases. However, given the patient's low thyroglobulin tumor marker level following surgery, alternative diagnoses were explored. The initial CT-guided biopsy of the patient's mediastinal mass was an FNA sample. No core biopsies were obtained. In addition, the washout from the FNA was not analyzed for thyroglobulin or PTH. This is a potential limitation of our initial work-up of the patient's abnormal finding on imaging. The patient's serum corrected calcium levels were normal at initial evaluation. The differential diagnoses ranged from acute or chronic inflammatory processes to benign or malignant lesions. Inflammatory lesions localized to the thorax include acute respiratory infections, pulmonary tuberculosis, pulmonary aspergilloma, myocardial infarction, and bronchiectasis. Benign and malignant lesions include mesothelioma, struma cordis, primary lung cancer, and breast cancer [2]. It is postulated that the pulmonary system is particularly susceptible to false positive I-131 surveys because of the demonstration of iodide secretion into the respiratory tract with elevated blood iodide levels [7].

Functional NIS-mediated radioiodine uptake has been documented in breast tissue, salivary glands, the gastrointestinal tract, and the thymus. There is an association with uptake into serous cavities or cysts such as scrotal hydroceles, lymphoepithelial cysts, ovarian cysts, renal cysts, and pleuropericardial cysts located in the anterior mediastinum [8].

Concentration of radioiodine by the thymus is not commonly observed by whole body scintigraphy, with estimates from a case series in a cohort of 175 female patients suggesting that approximately 1% to 2% of scans may demonstrate thymic uptake [9]. The normal pattern for physiological uptake of radioiodine by the thymus is either diffuse or in a dumbbell shape and is more prevalent in children. Uptake in adults can become more prominent following removal or ablation of the thyroidal tissues and with delayed imaging [2]. Increased thymic uptake has been shown in hyperplasia and carcinoma, but only rarely in cysts [10].

Here we present the rare finding of prominent radioactive iodine uptake by a thymic cyst following total thyroidectomy for papillary thyroid cancer. This patient raises a number of important concepts. First, there are multiple natural sites of radioiodine uptake beyond the thyroid. Second, following excision of a radioiodine avid neoplasm, secondary sites of uptake could represent metastatic disease. Third, there are anatomic variants with iodine avidity that should be considered in the differential diagnosis in cases of aberrant radioiodine uptake. Finally, one must consider the relatively low incidence of pulmonary metastases in DTC. In conclusion, clinicians should consider the array of differential diagnoses for lesions resembling pulmonary metastases from primary differentiated thyroid cancer.
